# In vitro differentiation of human skin-derived multipotent stromal cells into putative endothelial-like cells

**DOI:** 10.1186/1471-213X-12-7

**Published:** 2012-01-27

**Authors:** Radhakrishnan Vishnubalaji, Muthurangan Manikandan, May Al-Nbaheen, Balamuthu Kadalmani, Abdullah Aldahmash, Nehad M Alajez

**Affiliations:** 1 Stem Cell Unit, Department of Anatomy, College of Medicine, King Saud University, Riyadh 11461, Kingdom of Saudi Arabia; 2 KMEB, Department of Endocrinology, University of Southern Denmark, Odense, Denmark; 3 Department of Animal Science, School of Life Sciences, Bharathidasan University, Tiruchirappalli 620024, India

## Abstract

**Background:**

Multipotent stem cells have been successfully isolated from various tissues and are currently utilized for tissue-engineering and cell-based therapies. Among the many sources, skin has recently emerged as an attractive source for multipotent cells because of its abundance. Recent literature showed that skin stromal cells (SSCs) possess mesoderm lineage differentiation potential; however, the endothelial differentiation and angiogenic potential of SSC remains elusive. In our study, SSCs were isolated from human neonatal foreskin (hNFSSCs) and adult dermal skin (hADSSCs) using explants cultures and were compared with bone marrow (hMSC-TERT) and adipose tissue-derived mesenchymal stem cells (hADMSCs) for their potential differentiation into osteoblasts, adipocytes, and endothelial cells.

**Results:**

Concordant with previous studies, both MSCs and SSCs showed similar morphology, surface protein expression, and were able to differentiate into osteoblasts and adipocytes. Using an endothelial induction culture system combined with an in vitro matrigel angiogenesis assay, hNFSSCs and hADSSCs exhibited the highest tube-forming capability, which was similar to those formed by human umbilical vein endothelial cells (HUVEC), with hNFSSCs forming the most tightly packed, longest, and largest diameter tubules among the three cell types. CD146 was highly expressed on hNFSSCs and HUVEC followed by hADSSCs, and hMSC-TERT, while its expression was almost absent on hADMSCs. Similarly, higher vascular density (based on the expression of CD31, CD34, vWF, CD146 and SMA) was observed in neonatal skin, followed by adult dermal skin and adipose tissue. Thus, our preliminary data indicated a plausible relationship between vascular densities, and the expression of CD146 on multipotent cells derived from those tissues.

**Conclusions:**

Our data is the first to demonstrate that human dermal skin stromal cells can be differentiated into endothelial lineage. Hence, SSCs represents a novel source of stem/stromal cells for tissue regeneration and the vascularization of engineered tissues. Moreover, the CD146 investigations suggested that the microenvironmental niche might contribute to direct stromal cells multipotency toward certain lineages, which warrants further investigation.

## Background

There is growing need for novel technologies to restore, maintain, and enhance organ function. Since the 90s, stem cells have emerged as a new venue for regenerative medicine and tissue engineering. Human embryonic stem (ES) cells, induced pluripotent stem (iPS) cells and mesenchymal stem cells (MSCs), all has emerged as potential source for regenerative medicine and tissue engineering applications [1]. Among those, MSCs appear to have several advantages including the possibility of using autologous cells and the excellent safety record when transplanted into humans [2]. Currently, there is urgent need for engineered blood vessels to treat subjects with peripheral and coronary artery disease and for the vascularization of engineered tissues [3]. In general, MSCs have been isolated and characterized from different sources such as bone marrow [4], adipose tissue [4], umbilical cord blood [4], placenta [5], umbilical cord matrix [6], amniotic membrane [6] and dental pulp [7], and also been found in the stroma of various tissues and organs. Previous studies proved MSCs endothelial lineage differentiation and vascular potential [7-11]. Vasculogenesis and angiogenesis are the two major processes responsible for the development of blood vessels (i.e., neovascularization). The formation of endothelial tissue (vasculogenesis) is a course of action, referred to as the *in situ *formation of blood vessels from EPCs (endothelial progenitor cells) or angioblasts. These are differentiated from mesodermal cells and are prearranged to form a capillary network structure by growth and fusion of multiple blood islands [12,13]. Alternatively, angiogenesis will also result in new blood vessels arbitrated through the sprouting of new capillaries from pre-existing vessels, which happens in situations such as embryonic development [14,15].

Therapeutic angiogenesis is an essential process to maintain the integrity and treat disorders of insufficient perfusion of tissue by modulating the endothelial function or promoting blood vessels growth and proliferation. MSC-mediated vascular regeneration has been studied in vitro and *in vivo*, using angiogenic cytokines and growth factor supplements such as vascular endothelial growth factor (VEGF), basic fibroblast growth factor (bFGF) and hepatocyte growth factor (HGF) in attempt to enhance vasculogenesis when endogenous neovascularization is inadequate [16].

Several preclinical and clinical trials have demonstrated positive outcomes with MSCs in limb ischemia, ischemic stroke and peripheral ischemia with systemic sclerosis, spinal cord injury, and acute myocardial infarction [17-24]. In most of these cases, MSCs generated vascular network, reduced skin necrosis and restored the blood flow [17-24]. In another study, MSCs alone or co-cultured with EC promoted wound healing in diabetic and non diabetic animal models through the differentiation process and secretion of proangiogenic factors [25-30]. Hence, these investigations were indicative that, MSCs have paracrine effects through the secretion of a number of bioactive factors such as cytokines and growth factors that rejuvenate the injured or diseased tissues. Recently, MSC like population has been identified in skin dermis with immunoregulatory properties, multipotent differentiation into adipocyte, osteoblast, chondrocyte, neuron, hepatocyte and insulin-producing pancreatic cells (Table 1) [31-38]. These results suggest that, under the defined microenvironment, it is achievable to tailor the differentiation of MSC-like stromal cells into numerous types of cells for therapeutic applications. The angiogenic property of skin derived cells has been showed indirectly by previous studies, when co-cultured with EC. EC formed capillary-like tubes in response to paracrine factors secreted by skin cells, especially VEGF [39-41]. Furthermore, another study showed that the neosynthesis of extracellular matrix by skin cells is an important factor in in vitro angiogenesis [42]. Herein we report for the first time that SSCs have the potential to differentiate into endothelial-like cells and to form capillary network using an in vitro Matrigel (MG) angiogenesis assay. Using qRT-PCR and immunofluorescent imaging, differentiated cells expressed several markers (vWF, VEGFR, etc.) supporting their endothelial commitment. Our findings have significant implications for the utilization of SSCs as a novel source of multipotent cells for tissue engineering and regeneration.

**Table 1:** Multipotency of skin derived fibroblasts showed by previous studies

Name	Origins	Dissociation	Cultivation methods	Morphology	Expression of markers	Species	Multipotency (in vitro)	References
**Dermis-derived multipotent stem cells (DMCs)**	Newborn dermis	Enzymatic digestion	IMDM with 10 ml/L FBS and 100 U/mL penicillin and 100 microgram/mL streptomycin.	Adherent cells - spindle shaped	DMCs were positive for CD59, CD90, CD44, vascular cell adhesion molecule-1 (VCAM-1) and intercellular adhesion molecule-1 (ICAM-1). DMCs were negative for pan-cytokeratin, cytokeratin19, factor VIII, CD31, CD45, CD34, a-smooth muscle actin (a-SMA), desmin, collagen II and nestin. DNA microarray - G8Genes associated with pancreatic cells, transcripts for epithelial cell, neural cell, and hepatocyte as well.	Animal	Adipocyte, Osteoblast, Chondrocyte, Neuron and insulin- producing pancreatic cell.	Chun-Meng Shi et al (2004) [34]
**Skin derived pre cursors(SKPs)**	Neonatal mice	Trypsin and collagenase	S-MEM with 8% FBS treated with Chelex resin. 3D - culture done.	Adherent cells - spindle shaped	CD49f, CD49, CD117, CD34 were positive CD45 negative.	Animal	Adipocyte, chondrocyte, osteoblast and muscle	Crigler L et al (2007) [37]
**MSC like scalp-derived adherent cells (hSCPs)**	Scalp tissues	0.1% trypsin	DMEM-HG, 20% FBS with 20 ng/ml epidermal growth factor (EGF) and 20 ng/ml FGF2	Adherent cells - spindle shaped	CD44, CD49 (d, e, f), CD166, CD105, CD29, SH2, SH4, CDw90, EGFR, PDGFRa	Human	Adipocyte, Osteoblast, Chondrocyte and Neuron	Daniel Tzu-bi Shih et al (2005) [32]
**Human Fibroblasts equivalent to MSCs**	Apical skin	collagenase (1-2 mg/ml)	RPMI 1640 with 20% FCS, glutamine, penicillin and streptomycin	Adherent cells - spindle shaped	CD73, CD90, CD105, MHC class I and CD271 are positive. CD31, CD34, CD45, HLA DR, CD19, CD14 are negative	Human	Multipotency study not done but demonstrated fibroblasts are Immunoregulatory Cells and Functionally Equivalent to MSCs.	Muzlifah A. Haniffa et al (2007) [31].
**Multipotenet fibroblasts**	Foreskin	Collagenase	DMEM containing 10% FBS.	Adherent cells - spindle shaped	CD13 (62.1%), CD29 (53.2%), CD49d (38.4%), CD105 (30.9%), Stro-1 (8%) and a low level of CD34 (1.7%), negative for CD45, CD106 and CD133. Nestin negative, Vimentin positive.	Human	Adipocyte, Osteoblast, Chondrocyte, Neuron and Hepatocyte (6.4% population are tripotent),19.1% of clones were bipotent and 10,6% of the clones were unipotent.	Fu Guo Chen et al (2007) [33]
**Fibroblastic mesenchymal stem-cell-like cells**	Juvenile foreskin	0.075% collagenase	DMEM with GlutaMAX-I, 4.5 g ⁄ l glucose and pyruvate and 10% FBS.	Adherent cells - spindle shaped	CD14(-), CD29(+), CD31(-), CD34(-), CD44(+), CD45(-), CD71(+), CD73 ⁄ SH3-SH4(+), CD90 ⁄ Thy-1(+), CD105 ⁄ SH2(+), CD133(-) and CD166 ⁄ ALCAM(+). Expressed vimentin, fibronectin and collagen; they were less positive for a-smooth muscle actin and nestin, while they were negative for epithelial cytokeratins.	Human	Adipocyte and chondrocyte	Katrin Lorenz et al (2008) [36]
**Dermal mesenchymal stem cells**	Foreskin	Enzymatic digestion	clonal subcultivation in DMEM with meracaptoethanol	Adherent cells - spindle shaped	CD90 and CD105 were positive, CD34, c-kit and CD133 were negative	Human	Adipocyte, osteoblast and muscle	Bartsch G et al (2008) [38]
**Multipotent dermal fibroblasts**	Foreskin	Collagenase	DMEM-HG with 10% FBS, 300 μg/m1 L-glutamine, 50 μg/ml vitamin C, 100 U/ml penicillin and 100 μg/ml streptomycin.	Adherent cells - spindle shaped	_	Human	Adipocyte, neuron, hapatocyte and insulin-producing islet cells.	Dan Bi et al (2010) [35]

## Results

### Morphology and characterization of cultured cells

**Figure 1:**
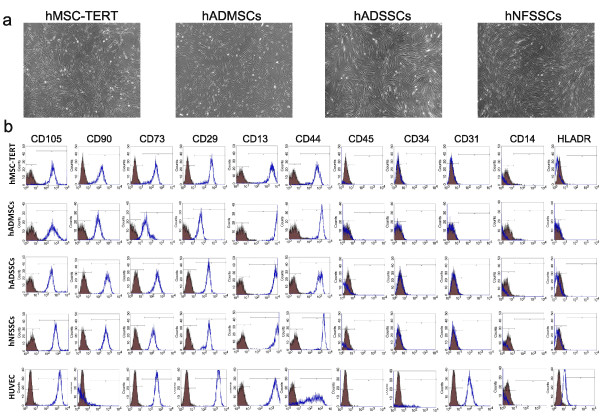
**Morphologic and phenotypic comparison of different cell types**. **(a) **The human bone marrow immortalized (hMSC-TERT line), adipose tissue (hADMSCs), and stromal cells derived from adult dermal (hADSSCs) and neonatal foreskin (hNFSSCs) cells were plastic adherent with a fibroblast-like spindle-shaped morphology (shown at magnification of 10X). **(b) **Flow cytometry analysis of cell surface protein expression of stromal cells, endothelial and hematopoietic lineage associated markers. Filled histograms represent cells stained by the corresponding isotype control antibody. Five thousand events were acquired for analysis.

The adherent cultured human MSC-TERT, ADMSCs, ADSSCs and NFSSCs exhibited fibroblast-like, spindle-shaped morphology when observed under light microscope (Figure 1a). Nonetheless, all groups were positive (> 90%) for stromal cell-associated markers (CD105, CD90, CD73, CD29, CD13, and CD44), and were negative (< 1%) for endothelial and hematopoietic lineage markers (CD45, CD34, CD31, CD14, and HLA DR) (Figure 1b), indicating great similarities between MSCs and SSCs. HUVEC expressed high levels (> 90%) of CD105, CD73, CD29, CD13, CD44 and CD31, and were dim positive for CD34 (> 7%) and HLADR (> 20%), and were negative for CD90, CD45 and CD14 (Figure 1b). The endothelial differentiation experiments were conducted on hADMSCs, hADSSCs and hNFSSCs at passage 4; while the immortalized hMSC-TERT cells were acquired at passage 47.

### Adipogenic and osteogenic differentiation potential

**Figure 2:**
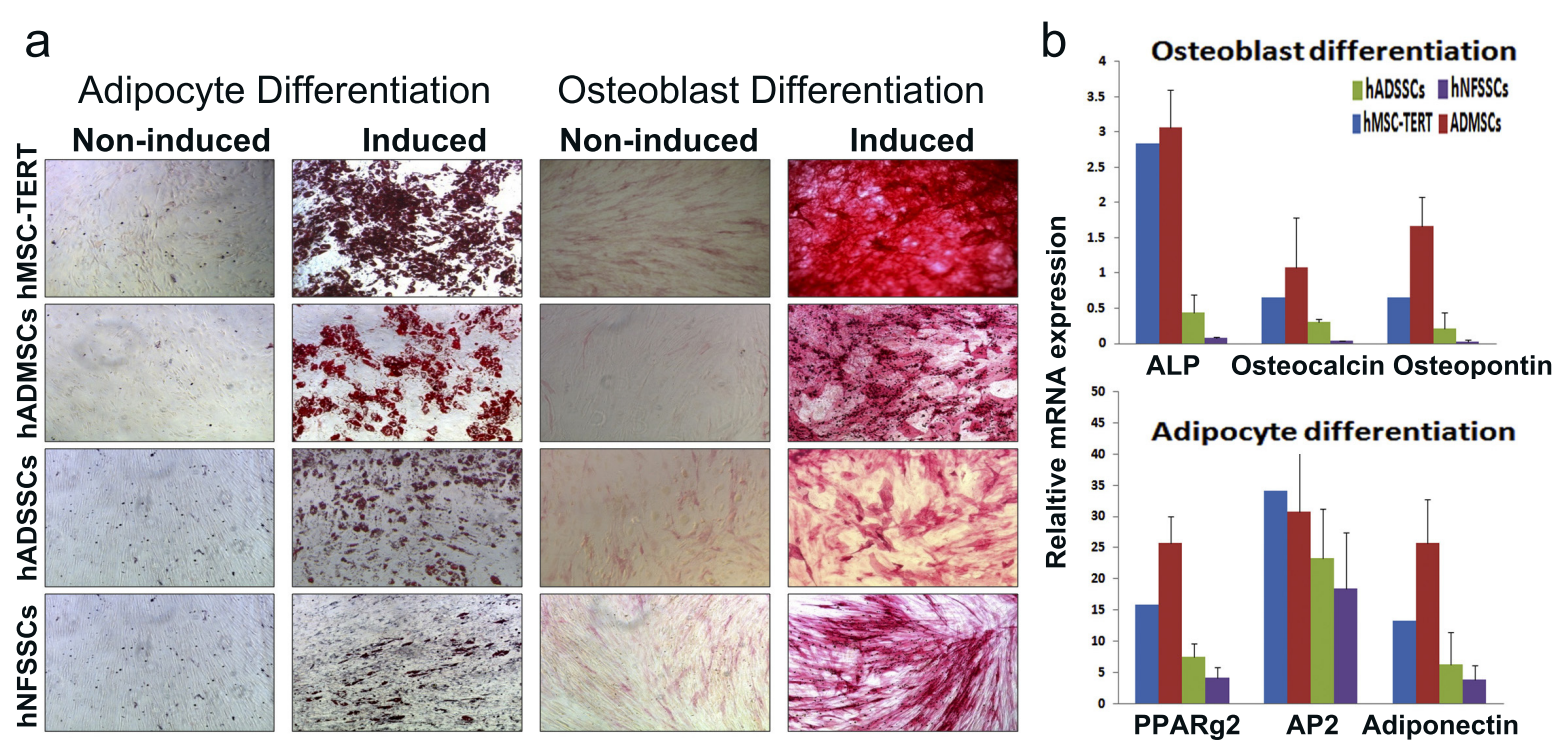
**(a) Multilineage differentiation potential of hMSC-TERT, hADMSCs, hADSSCs and hNFSSCs**. Cells were induced for 15 days under osteogenic conditions then were assessed for alkaline phosphatase activity (ALP) to measure osteogenesis, while cells induced for 15 days under adipogenic conditions were assessed by the development of neutral lipid vacuoles stainable with Oil Red O (shown at magnification of 10×). **(b) **Quantitative real-time PCR (qRT-PCR) analysis of adipocyte and osteoblast-associated gene expression in induced cells. After differentiation for 15 days, cells were harvested, and total RNA was extracted and the expression of adipocyte and osteoblast-associated genes were quantified. Gene expression was normalized to beta-actin and is presented as fold change compared to control (non-induced cells). Data are shown as mean ± S.D of at least two independent experiments, n = 5 (except hMSC-TERT cell line).

We assessed the differentiation potential of cultured MSCs and SSCs. When induced in adipogenic medium for 15 days, hMSC-TERT, hADMSC, hADSSCs, and hNFSSCs began to accumulate intracellular lipid vacuoles that progressively filled the cytoplasm adjacent to the cell membrane, and were positive for Oil Red O staining, confirming their adipogenic phenotype (Figure 2a). Similarly, cells induced under osteogenic conditions exhibited positive alkaline phosphatase (ALP) staining, thus confirming their osteogenic differentiation. By contrast, non-induced control cells didn't reveal any positive staining. Notably, hADSSCs and hNFSSCs cells demonstrated less osteogenic and adipogenic differentiation potential when compared to MSCs (Figure 2a and 2b).

### Differentiation of MSCs and SSCs into Endothelial Cells (EC)

**Figure 3:**
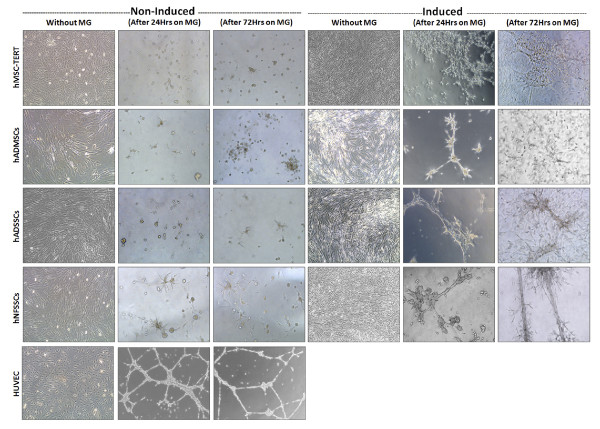
**In vitro angiogenic potential of MSCs and SSCs**. Phase contrast image analysis of induced and non-induced hMSC-TERT, hADMSCs, hADSSCs, hNFSSCs and HUVEC on semisolid medium (Matrigel; MG) in the presence and absence of endothelial growth supplements. Morphological changes were observed at different time points (0, 24 and 72 h) post culture on matrigel. Images are shown at magnification of 10×. Data are representative of at least two independent experiments.

**Figure 4:**
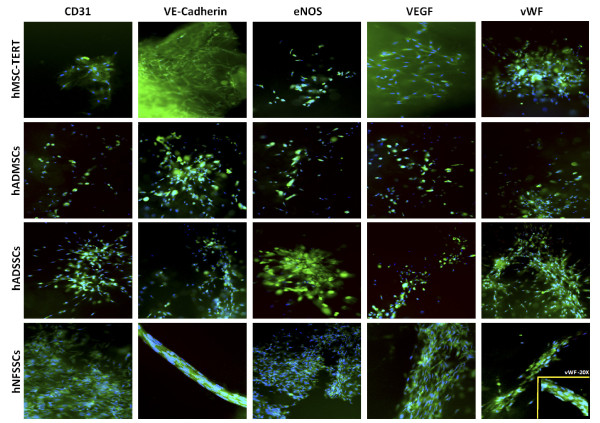
**Immunofluorescence staining for endothelial-associated markers 72 h post-induction on matrigel**. hMSC-TERT, hADMSCs, hADSSCs and hNFSSCs were induced for 7 days and then were plated on matrigel-coated wells. Expression of endothelial associated markers (CD31, VE-cadherin, eNOS, VEGF165 and vWF) was assesses using immunofluorescence microscopy. 4,6-Diamidino-2 phenylindole (DAPI) was used to counter stain for cell nuclei (images are shown at magnification of 10×). Notably, hNFSSCs exhibited tremendous tightly packed capillary tube-like structures. Lower right panel is a close-up for vWF staining shown at 20× magnification.

**Figure 5:**
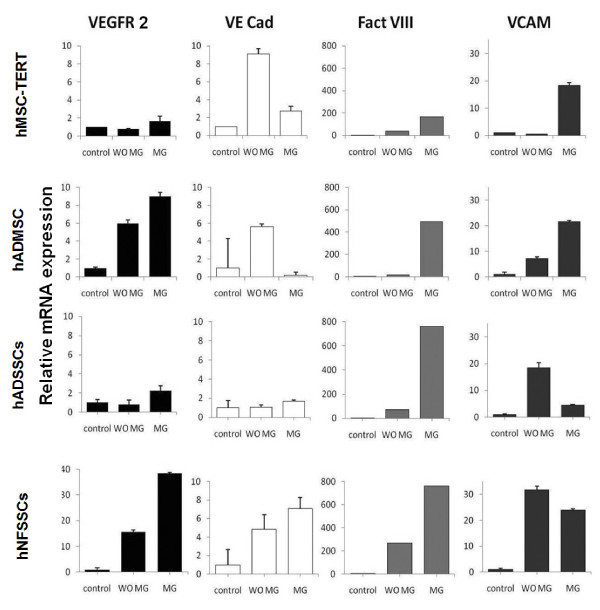
**Quantitative real-time PCR (qRT-PCR) analysis of endothelial-associated gene expression in induced and non-induced cells**. After endothelial differentiation for 7 days, cells cultivated on matrigel (MG) and without matrigel (WO MG) for additional 3 days were harvested, and total RNA was extracted and the expression of endothelial and angiogenesis-associated genes were quantified. Gene expression was normalized to beta-actin and is presented as a fold change over non-induced (control) cells. Data are shown as mean ± S.D of at least two independent experiments, n = 2.

In order to assess the endothelial differentiation potential of hMSC-TERT, hADMSCs, hADSSCs and hNFSSCs, cells were induced for 7 days as described in materials and methods. As shown in Figure 3, cell morphology of induced cultures (without MG) did not show significant difference when compared to non-induced (without MG) culture conditions, all maintaining fibroblast-like spindle shaped morphology. Nonetheless, induced cultures exhibited similar expression pattern of stromal lineage CD markers to those seen under non-induced conditions (Additional file 1: Figure S1 and Figure 1). The angiogenic capability of induced cells was then assessed using an in vitro endothelial tube formation assay, whereas HUVEC cells were used as positive control. Induced cells were cultured in the presence of endothelial differentiation cocktail for 7 days, followed by migration to matrigel-coated plates. Twenty four hours later, cells exhibited tube-like interconnected structures, whereas the percentage of capillaries gradually increased until discrete matrigel regions were enclosed by cell islets or tubes (Figure 3, right), which was similar to capillary structures formed by HUVEC (Figure 3, bottom left). In contrast, non-induced cells on matrigel showed scattered populations with very few tube-like clusters, mainly in the hNFSSCs and hADSSCs (Figure 3). Notably, hNFSSCs exhibited the highest tube formation capabilities compared to the other cell types when cultured on matrigel (Figure 3). To confirm the endothelial differentiation under our culture conditions, induced cells on matrigel were stained for CD31, VE-cadherin, eNOS, VEGF165 and vWF and were visualized under fluorescent microscope. Non-induced cells did not express any of these markers, even when cultured on matrigel (data not shown). On the other hand, cells induced for 7 days and cultured on matrigel exhibited significant expression of the aforementioned endothelial markers (Figure 4). Concordant with the capillary formation data from Figure 3, hNFSSCs and hADSSCs exhibited the highest expression level of endothelial-associated markers (Figure 4). To further confirm their endothelial differentiation, the expression levels of various endothelial and angiogenesis-associated genes (Vascular endothelial growth factor receptor 2 (VEGFR2; KDR/flk1), Vascular endothelial cadherin (VE-Cadherin; CD144), Factor VIII (FVIII; AHF) and Vascular cell adhesion molecule 1 (VCAM1; CD106)) was assessed in non-induced, induced, and induced plus matrigel cultured cells using quantitative real-time PCR. The data presented in Figure 5 demonstrated significant increases in the expression of VEGFR2, VE-cadherin, FVIII, and VCAM1 under induction culture conditions, further supporting their endothelial commitment.

### Correlation between vascular density, CD146 expression and endothelial differentiation

**Figure 6:**
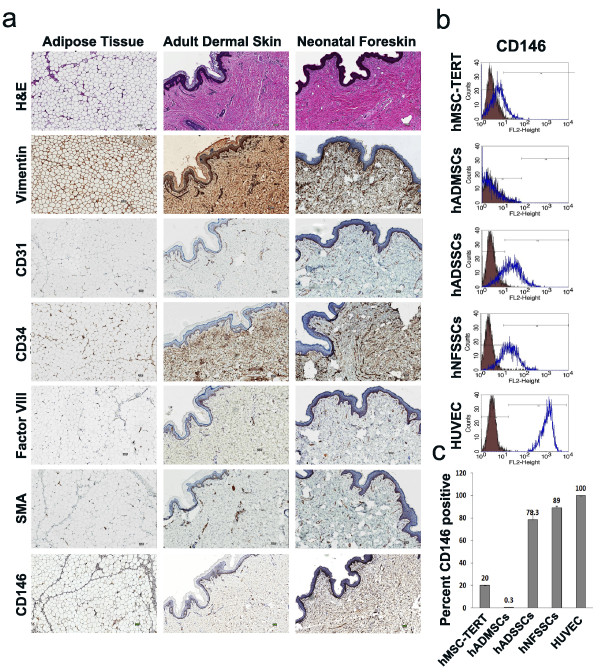
**Comparative vasculature and CD146 analyses of hMSC-TERT, hADMSCs, hADSSCs, hNFSSCs and HUVEC**. **(a) **Hematoxylin-and eosin (H&E), Vimentin, CD31, CD34, Factor VIII, CD146 and smooth muscle actin (SMA) staining of human adipose, adult dermal and neonatal foreskin FFPE tissue sections. Scale Bar = 50 micron. **(b) **Flow cytometry analysis of CD146 protein surface expression on hMSC-TERT (*n *= 1), hADMSCs (*n *= 6), hADSSCs (*n *= 6), hNFSSCs (*n *= 5), HUVEC (*n *= 1). Filled histograms represent cells stained with the corresponding isotype control antibody. **(c) **Quantitative presentation of the percentage of CD146^+ ^population in different cell types.

Interestingly, immunohistological examination (CD31, CD34, Factor VIII, CD146 and smooth muscle actin (SMA) [11,43]) of adipose, adult dermal skin, and neonatal foreskin tissues revealed a higher vascular density in human neonatal foreskin, followed by dermal skin and adipose tissue (Figure 6a), which apparently paralleled the endothelial differentiation potential of cells derived from those tissues, suggesting that hNFSSCs and hADSSCs might be "more committed" toward endothelial differentiation. When we examined CD146 expression by FACS, surprisingly it was predominantly present on hNFSSCs (> 89%) and hADSSCs (> 78%), which was close to its expression o HUVEC (100%), while it was expressed at much lower level on hMSC-TERT (> 20%) and hADMSCs (< 1%) (Figure 6b and 6c). Similarly, immunohistochemical staining of CD146 revealed the same expression trend when comparing adipose, adult dermal and neonatal foreskin tissues (compare Figure 6a and 6b).

## Discussion

In tissue engineering, development of blood vessel is one of the most attractive areas of research for the treatment of vascular diseases and tissue vascularization [43,44]. Due to the differentiation and proliferation capabilities of MSCs, this cell population is currently an integral part of regenerative medicine [4,45,46]. ECs are the main element of a primitive vascular plexus, however, the communication between ECs and other cells such as pericytes is also essential for vasculogenesis. VEGF act as a potential regulator of EC-pericyte interaction and vascular progenitor cell differentiation in early embryogenesis. Although embryonic stem cells have been successfully differentiated into endothelial cells in vitro [47], the use of ES for regenerative medicine is still controversial. On the other hand, adult blood vessel-derived ECs have recently been identified for their capability to form three dimensional vessel-like structures through in vitro EC co-culture system [48]. Their inadequate in vitro proliferation had limited the wide utilization of ECs in tissue engineering [49]. Considering procurement risk and their short lifespan, researchers have been searching for alternative sources of multipotent stem cells capable of differentiation into endothelial lineage [50-53]. Thereafter, EC-like cells differentiated from UCB (umbilical cord blood), placenta, umbilical cord Wharton's jelly and adipose tissue derived MSCs have been reported [8-11]. Recently, it was shown that skin derived cells have adipogenic and osteogenic differentiation potential [54]. Nonetheless, a number of other studies revealed that dermal skin cells are multipotent and are capable of differentiation into neurons, hepatocytes, and insulin-producing pancreatic-like cells [33,34]. Consistent with previous studies, herein we demonstrated that both adult and neonatal stromal cells isolated from adult dermal skin and neonatal foreskin have similar phenotype to BM and adipose-derived MSCs and could be differentiated into adipogenic and osteogenic lineages under the proper induction conditions [33,34,37].

More importantly, our current study demonstrated for the first time that human adult dermal and neonatal foreskin derived CD13^+ ^CD29^+ ^CD44^+ ^CD73^+ ^CD90^+ ^CD105^+ ^stromal cells are capable of differentiating into endothelial cells and forming CD31^+ ^VEGF^+ ^VE-cadherin^+ ^eNOS^+ ^vWF^+ ^capillary tube-like structures.

Abdallah et al. previously reported that hMSC-TERT cells can undergo endothelial differentiation as evident by the upregulation of VEGFA, EPAS-1, HIF-2a (hypoxia-inducible transcriptional factor-2), ETB (endothelin receptor type B), while no change in VEGFR gene expression was observed after 3 and 7 days of induction [55]. When cultured on matrigel, induced hMSC-TERT stained positive for vWF, which collectively would be consistent with the results obtained in the current study. Our histological data revealed that hNFSSCs and hADSSCs are derived from a vascular rich anatomical regions, compared to hADMSCs (Figure 6a), suggesting that the differentiation potency might depend on the tissue from which the cells were derived. When compared to hMSC-TERT and hADMSCs, hNFSSCs and hADSSCs demonstrated the highest formation of tightly-packed capillary tube-like networks in an in vitro angiogenesis assay. Interestingly and in contrast, hNFSSCs and hADSSCs showed the least osteogenic and adipogenic differentiation potential compared to hMSC-TERT and hADMSCs (Figure 2a and 2b), again suggesting that the majority of skin-derived stromal cells might already be committed toward endothelial differentiation to better serve their anatomical location, especially in the event of wound healing which requires rapid neovascularization. Concerning basal MSC characterization, all cells isolated from these four sites exhibited typical MSC characteristics: a fibroblastoid morphology, expression of a typical set of surface markers (CD13^+ ^CD29^+ ^CD44^+ ^CD73^+ ^CD90^+ ^CD105^+)^, and lack of the expression of endothelial and hematopoietic markers (CD34^-^, CD31^-^, CD14^-^, CD45^-^, HLA-DR^-^). Therefore, it is unlikely that our culture system expanded pre-existing endothelial progenitors as the flow cytometry data clearly demonstrated homogeneous population of fibroblast-like spindle-shaped cells which did not express CD34 and CD31 endothelial progenitor markers [56,57]. Interestingly, our data suggested a possible correlation between CD146 expression and endothelial differentiation potential (compare Figure 6b and Figure 3). Consistent with this, recent report has found that CD146^+ ^MSCs within bone marrow are localized in the perivascular region, while the CD146^- ^cells were more in the bone lining region, again suggesting a possible correlation between CD146 expression on hMSCs and hSSCs and vascular commitment [58]. Currently, CD146 is regarded one of the commonly reported positive surface antigens of MSCs, among the several stem cell associated markers [59]. On the other hand, CD146 is also considered as an endothelial and pericyte marker [60]. Therefore, our data suggest that stromal cell populations that are highly positive for CD146 might be more inclined to generate endothelial cells, however this assumption warrants further investigation.

## Conclusions

Our results indicate that SSCs derived from human adult and neonatal skin, are multipotent that have the potential to differentiate into endothelial-like cells, in addition to their adipocytes and osteoblasts differentiation capabilities. Therefore, our data is the first to demonstrate endothelial-differentiation potential of dermal-skin stromal cells, which potentially could have a myriad of implications toward understanding the basic biology of wound healing, in tissue engineering, and in regenerative medicine applications.

## Methods

This project was approved by the Institution Review Board of King Saud University Medical College and Hospital (10-2815-IRB).

### Cell culture

Cells were cultured in Dulbecco's Modified Eagle Medium (DMEM) supplemented with D-glucose 4500 mg/L, 4 mM L-Glutamine and 110 mg/L Sodium Pyruvate, 10% Fetal Bovine Serum (FBS), 1x penicillin--streptomycin (Pen-strep) and Non essential amino acids (all purchased from Gibco-Invitrogen, USA). hMSC-TERT (bone marrow immortalized cell line) was kindly donated by Dr. Mosthafa Kassem, Department of Endocrinology and Metabolism, University Hospital of Odense, Odense, Denmark [61]. Human umbilical vein endothelial cells (HUVEC) were purchased from Lonza biotech (CC-2517; Walkersville, USA), it was cultured in vascular cell basal medium (ATCC; PCS-100-030) supplemented with microvascular endothelial growth factors (ATCC; PCS-100-041). Adipose tissue and adult dermal skin were received from patients undergoing abdominal bariatric surgery, lipectomy, knee replacement or gastro intestinal operations. Neonatal foreskins were received from voluntary circumcisions of 2-3 days male babies. All donors and/or their parents gave written informed consent for the use of their tissues for scientific purposes. All tissues were washed 3 times with PBS contain 1x Pen-Strep. The epidermis was manually removed from skin, and the dermis was cut into 1-3 mm pieces, placed in 3 cm culture dishes where epidermis layer facing up wards and the dermis layer contacting the culture surface with few drops of culture medium. Tissues were incubated at 37°C and 5% CO_2 _in a humidified environment. After few hours, and once the tissues were attached, the level of culture medium was raised and culture was maintained for a week or until the outgrowth of fibroblast-like spindle shaped cells was visible. Adipose tissues were minced mechanically then incubated in 1% Collagenase type I (Gibco-Invitrogen, USA) for 45 min with gentle agitation at 37°C. After inactivation of collagenase by culture medium DMEM, debris were separated from pellets of stromal vascular fraction (SVF) by centrifugation at 500 g for 15 min. SVF cells were resuspended in culture medium and plated in 25 cm^2 ^tissue culture flask and maintained in a humidified incubator at 37°C and 5% CO_2_. The next day, all non adherent cells were removed by washing. In few days, the growth of MSCs was visualized under an inverted microscope. Cells were fed with fresh medium every 3-4 days until cells reached 70-80% confluency. Residual skins were removed from explant cultures and adherent cells were passaged from skin and SVF culture by standard trypsinization method (Trypsin-EDTA (0.05%); Gibco-Invitrogen, USA).

### Histological examination

The Formalin-Fixed Paraffin-Embedded human adipose tissue, neonatal and adult skins were stained according to the manufacturer's staining protocol on a Bond-max™ fully automated IHC & ISH staining system (Leica Microsystems GmbH, Germany) and then stained with hematoxylin and eosin (H&E) and Vimentin (clone V9; 1:50), CD31 (clone 1A10; 1:50), CD34 (clone QBend/10; 1:50), Factor VIII (clone 36B11; 1:100), CD146 (abcam, USA; clone P1H12; 1:100) and smooth muscle actin (SMA; clone alpha SM-1; 1:50) using manufacturer's standard protocols (except CD146 all antibodies from Leica Biosystems Newcastle Ltd; UK). Slides were digitalized using High-resolution whole-slide digital ScanScope scanner (Aperio Technologies, Inc.). The digital slide images were then viewed and analyzed using the viewing and image analysis tools of Aperio's ImageScope software (Aperio Technologies, Vista, CA, USA).

### Endothelial cell differentiation

To assess the endothelial differentiation potential, hMSC-TERT, ADMSCs, hNFSSCs and hADSSCs were cultured in 25 cm^2 ^tissue culture flasks. When cells reached 70-90% confluency, medium was replaced with endothelial medium DMEM + 2% FBS, 1% Pen-strep, 50 ng/mL VEGF (R&D systems, USA), 10 ng/mL bFGF (Sigma-Aldrich, USA) and 50 μg/mL ECGS (endothelial cell growth supplement, BD Bioscience, USA) for 7 days. Medium was changed every 2 days.

### Endothelial cell tube formation (*in vitro *angiogenesis)

In vitro matrigel angiogenesis assay was utilized to assess the tube-formation capabilities of hMSC-TERT, hADMSCs, hADSSCs, and hNFSSCs under normal and endothelial culture conditions. Matrigel matrix was thawed on ice at 4°C overnight, then 0.3 ml of chilled matrigel solution (10 mg/mL, Basement Membrane Matrix, BD Bioscience) was applied to one well in a 24-well plate using ice-cooled tips and incubated for 1 h at 37°C. After 7 days of endothelial inductions, both control and induced cells were trypsinized and platted on top of the matrigel-coated 24-well plates (2 × 10^4 ^cells per well) and were further incubated at 37°C in a 5% CO_2 _humidified atmosphere for 1-3 days. Tube formation assay was carried out along with HUVEC as a positive control. Tube formation was examined using an inverted phase-contrast microscope Carl Zeiss--Axio observer.1 equipped with a digital camera (Axiocam MRc5).

### Immunophenotyping by flow cytometry

HUVEC and cells from induced and non-induced cultures (after 7 days) were harvested using 0.05% trypsin-EDTA and were washed twice in ice-cold PBS supplemented with 0.5% BSA and resuspended at 10^6 ^cells per ml. Ten microliter of PE-conjugated mouse anti-human CD146, CD73, CD29 and HLA-DR, FITC-conjugated mouse anti-human CD34, CD90, CD45, CD13, CD184 and CD31, or APC-conjugated mouse anti-human CD105, CD14 and CD44 antibodies (all from BD Biosciences, except the anti-human CD105, which was purchased from R&D systems) was added to 100 μl of cell suspension (10^5 ^cells). Negative control staining was performed using a FITC, PE, or APC-conjugated mouse IgG1 isotype control antibodies, respectively. Cells were incubated for 30 min at 4°C in dark, then were washed with PBS to remove excess antibodies, and then were resuspended in 500 μL of PBS and were analyzed using BD FACS Calibour flow cytometer (BD Biosciences). Living cells were gated in a dot plot of forward vs. side scatter signals obtained on linear scale. At least, 5,000 gated events were acquired on a Log fluorescence scale. Data were analyzed using Cell Quest Pro Software Version 3.3 software (BD Biosciences).

### Immunofluorescence staining

Adherent cells were fixed with 4% cold paraformaldehyde (Sigma) for 15 min and permeabilized with 0.1% Triton X-100 (Sigma) for 10 min. After washing with PBS, cells were blocked with 3% bovine serum albumin (BSA, Sigma) for 30 min, followed by incubating with primary antibodies against CD31 (5 μg/mL) VE-Cadherin (BV9; 1/25), eNOS (Endothelial Nitric Oxide Synthase; 5 μl/mL), vWF (von Willebrand Factor; 4 F9; 10 μl/mL) all are from Abcam (USA) and VEGF (VEGF 165; 8 μg/mL; US Biological) at 4°C overnight. After removal of primary antibodies, cells were washed three times with PBS, and the FITC-labeled secondary antibody (goat polyclonal to mouse or rabbit IgG; both 1/4000; Abcam) was added and incubated for 1 h at room temperature. Cells were washed three times with PBS and counter stained with DAPI (4',6-diamidino-2-phenylindole) nuclear dye and were observed under Leica DM5000 B fluorescence microscope.

### Osteogenic differentiation

Cells were seeded at a density of 0.05 × 10^6 ^cell/ml in 6 well plates (for cytochemical staining and RNA isolation) and were grown for 24 h in standard DMEM growth medium. At 70-80% confluence, the medium was replaced in test wells by osteogenic induction medium supplemented with DMEM containing 10% FBS, 1% Pen Strep,50 μg/mL L-ascorbic acid (Wako Chemicals GmbH, Neuss, Germany), 10 mM β-glycerophosphate (Sigma), and 10 nM calcitriol [(1α,25-dihydroxy vitamin D3) (sigma)], 10 nM Dexamethasone (Sigma). The osteogenic medium was changed every 3 days and the experiments were terminated at day 15. Cells cultured in the regular culture medium were considered as experiment control.

### Adipocytic differentiation

Cells were seeded at a density of 0.05 × 10^6 ^cell/ml in 6 well plates (for cytochemical staining and RNA isolation) and were grown for 24 h in standard DMEM growth medium. At 90-100% confluence, the medium was replaced in all test wells with adipogenic induction medium supplemented with 10% FBS and adipogenic-induction mixture (AIM) containing 10% Horse Serum (Sigma), 1% Pen-strep, 100 nM dexamethasone, 0.45 mM isobutyl methyl xanthine [(IBMX) (Sigma)], 3 μg/mL insulin (Sigma), and 1 μM Rosiglitazone [(BRL49653) (Novo Nordisk, Bagsvaerd, Denmark)]. The adipogenic medium was replaced every 3 days, and experiments were terminated at day 15. Cells cultured in regular medium were considered as experiment control.

### Alkaline phosphatase (ALP) staining for osteoblasts

Cells were induced to osteoblasts differentiation for 15 days as described above, then cells were washed in PBS twice, fixed in acetone/citrate buffer 10 mM (1.5: 1) at pH 4.2 for 5 min at room temperature and incubated with alkaline phosphatase (ALP) substrate solution (naphthol AS-TR phosphate (Sigma) prepared 1:5 in water plus 10 mg Fast red TR (Sigma), in 24 mL of 0.1 M Tris buffer, pH 9.0) for 1 h at room temperature. Cells were rinsed with water, stored in PBS and photographed using Carl Zeiss--Axio observer.1 equipped with a digital camera (Axiocam MRc5).

### Oil red-O staining for adipocytes

Oil red-O stain was utilized to assess the accumulation of cytoplasmic lipid droplets. Cells were differentiated into adipocytes for 15 days, washed twice in PBS, fixed in 4% formaldehyde for 10 min at room temperature, rinsed once in 3% isopropanol, and stained for 1 h at room temperature with filtered Oil red-O staining solution (prepared by dissolving 0.5 g Oil red-O powder in 60% isopropanol). Cells were rinsed with water then photographed using Carl Zeiss--Axio observer.1 equipped with a digital camera (Axiocam MRc5).

### Quantitative real-time PCR for gene expression

To measure the expression level of endothelial, adipocyte and osteoblast-associated genes, total RNA was isolated using the FastLane cDNA kit (Qiagen) according to manufacturer's instructions. Cell lysate containing RNA from two independent treatments was combined and quantitated. All samples were normalized to the lowest concentration of RNA obtained. Complementary DNA (cDNA) was synthesized from 4 μL of the normalized RNA samples using FastLane cDNA kit according to the manufacturer's instructions. Relative levels of mRNA were determined from cDNA by real time PCR (Applied Biosystem-Real Time PCR Detection System) with QuantiFast SYBR Green PCR kit (Qiagen) according to the manufacturer's instructions.

### Endothelial-associated genes

The sequence for PCR primers (all from Invitrogen limited, UK) were as follows (5'-3'): VEGFR2 (KDR/flk1; Fw: CTTACCCCAGGATATGGAG and Rev: CCGTCAAGGGAAAGACTACG), VE-Cadherin (CD144; Fw: CCCGTCTTTACTCAATCCACA and Rev: GGGTTTGATGATACCCTCGTT), FVIII (AHF; Fw: GTTCGTCCTGGAAGGATCGG and Rev: CACTGACACCTGAGTGAGAC) and VCAM-1 (CD106; Fw: TCTCATTGACTTGCAGCACC and Rev: TTCTTGCAGCTTTGTGGATG), β-actin (Fw: AGCCATGTACGTTGCTA and Rev: AGTCCGCCTAGAAGCA).

### Adipocyte and osteoblast-associated genes

The sequence for PCR primers (all from Invitrogen limited, UK) were as follows (5'-3'): PPAR-*γ *2 (Fw: CTCCACTTTGATTGCACTTTGG and Rev: TTCTCCTAT TGACCCAGAAAGC), aP2 (Fw: TGGTTGATTTTCCATCCCAT and Rev: GCCAGGAATTTGACGAAGTC), Adiponectin (Fw: ATGTCTCCCTTAGGACCAATAAG and Rev: TGTTGCTGGGAGCTGTTCTACTG), ALP (Fw: ACGTGGCTAAGAATGTCATC and Rev: CTGGTAGGCGATGTCCTTA), Osteocalcin (Fw: AGAGCGACACCCTAGAC and Rev: CATGAGAGCCCTCACA) and Osteopontin (Fw: GGTGATGTCCTCGTCTGTA and Rev: CCAAGTAAGTCCAACGAAAG). The relative abundance of target mRNA was quantified relative to the control β-actin gene expression from the same reaction setup, using the 2^-ΔΔCT ^method [62].

## Abbreviations

**HUVEC**: Human umbilical vein endothelial cells; **SSCs**: Skin stromal cells; **hNFSSCs**: Human neonatal foreskin stromal cells; **hADSSCs**: Human adult dermal skin stromal cells; **hMSC-TERT**: Human mesenchymal stem cell--telomerase reverse transcriptase (immortalized cell line); **hADMSCs**: Human adipose derived mesenchymal stem cells; **CD**: Cluster of differentiation; **RT-PCR**: Real time--polymerase chain reaction.

## Competing interests

The authors declare that they have no competing interests.

## Authors' contributions

RV design and carried out all the experimental work and wrote the manuscript. MM supported for FACS and immunofluorescence sample preparation and histology experiment. NMA conceived the study, edited and finalized the manuscript. BK gave advice and participated in its design. AA and MAN participated in its design, coordination and obtained funding. All authors read and approved the final manuscript.

## Supplementary Material

Additional file 1 **Figure S1**. Flow cytometry analysis of stromal associated markers on induced cells. All the groups expressed CD13, CD29, CD44, CD73, CD90, and CD105. Filled histograms represent cells stained by the corresponding isotype control antibody. Five thousand events were acquired for analysis. Click here for file
