# Supplementary material for: In vitro differentiation of human skin-derived multipotent stromal cells into putative endothelial-like cells
Source: BMC Dev Biol. 2012 Jan 27;12:7. doi: 10.1186/1471-213X-12-7 (PMC3280173; doi:10.1186/1471-213X-12-7)
Supplement: Additional file 1 — Figure S1. Flow cytometry analysis of stromal associated markers on induced cells. All the groups expressed CD13, CD29, CD44, CD73, CD90, and CD105. Filled histograms represent cells stained by the corresponding isotype control antibody. Five thousand events were acquired for analysis. [file 1471-213X-12-7-S1.PDF]

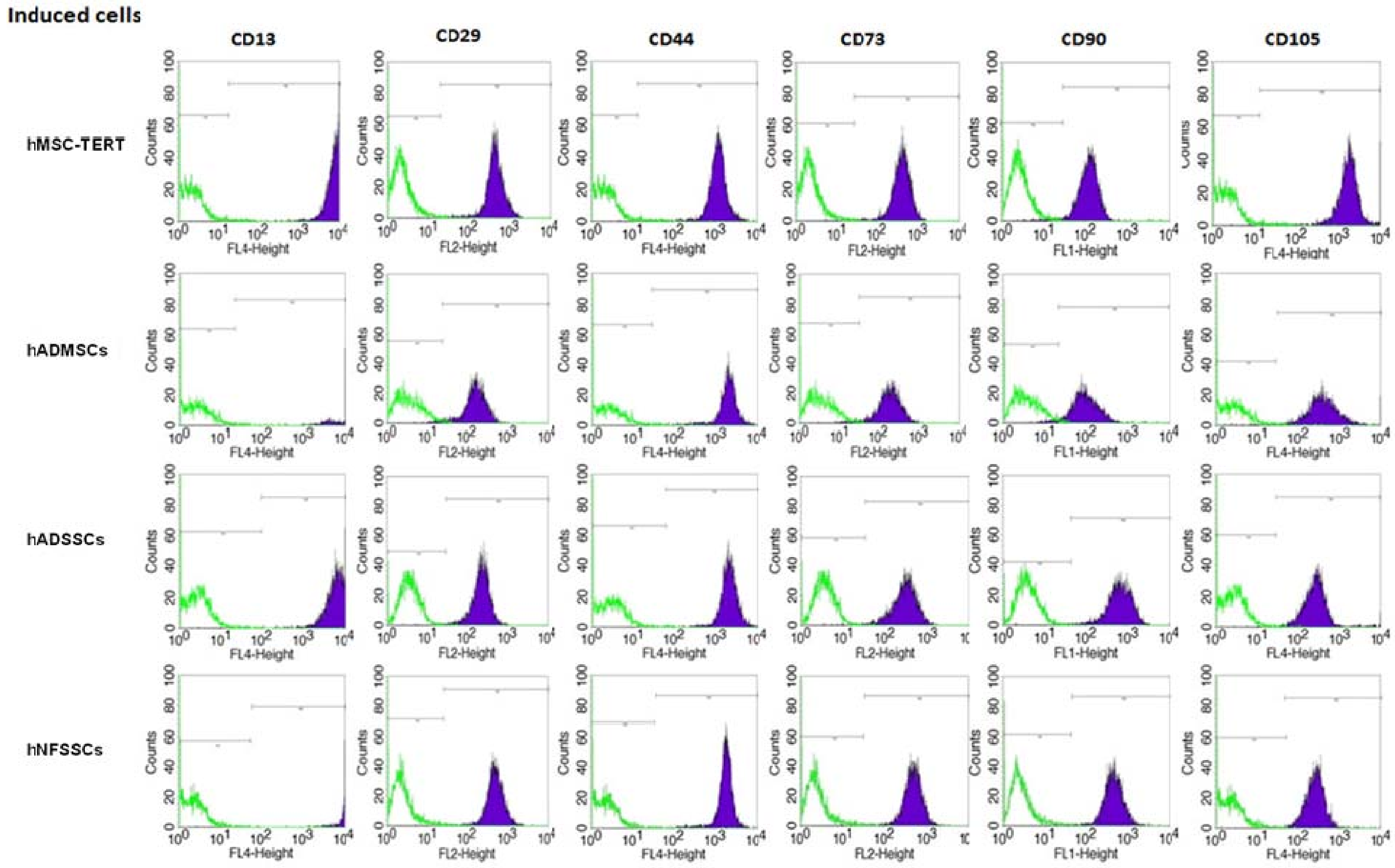


**Supplementary figure 1.** Flow cytometry analysis of stromal associated markers on induced cells. All the groups express CD13, CD29, CD44, CD73, CD90, and CD105. Filled histograms represent cells stained by the corresponding isotype control antibody. Five thousand events were collected for analysis.
